# Cement-Based Electrochemical Systems for Structural Energy Storage: Progress and Prospects

**DOI:** 10.3390/ma18153601

**Published:** 2025-07-31

**Authors:** Haifeng Huang, Shuhao Zhang, Yizhe Wang, Yipu Guo, Chao Zhang, Fulin Qu

**Affiliations:** 1 School of Civil Engineering, Southwest Jiaotong University, Chengdu 610031, China; douhuang@126.com (H.H.); 18352861265@163.com (S.Z.); 2 Centre for Infrastructure Engineering and Safety, School of Civil and Environmental Engineering, University of New South Wales, Kensington, NSW 2052, Australia; yizhe.wang3@student.unsw.edu.au (Y.W.); yipu.guo@student.unsw.edu.au (Y.G.); 3 Department of Civil and Environmental Engineering, The Hong Kong University of Science and Technology, Clear Water Bay, Hong Kong, China

**Keywords:** cement-based batteries, multifunctional materials, structural health monitoring, energy storage, smart infrastructure

## Abstract

Cement-based batteries (CBBs) are an emerging category of multifunctional materials that combine structural load-bearing capacity with integrated electrochemical energy storage, enabling the development of self-powered infrastructure. Although previous reviews have explored selected aspects of CBB technology, a comprehensive synthesis encompassing system architectures, material strategies, and performance metrics remains insufficient. In this review, CBB systems are categorized into two representative configurations: probe-type galvanic cells and layered monolithic structures. Their structural characteristics and electrochemical behaviors are critically compared. Strategies to enhance performance include improving ionic conductivity through alkaline pore solutions, facilitating electron transport using carbon-based conductive networks, and incorporating redox-active materials such as zinc–manganese dioxide and nickel–iron couples. Early CBB prototypes demonstrated limited energy densities due to high internal resistance and inefficient utilization of active components. Recent advancements in electrode architecture, including nickel-coated carbon fiber meshes and three-dimensional nickel foam scaffolds, have achieved stable rechargeability across multiple cycles with energy densities surpassing 11 Wh/m^2^. These findings demonstrate the practical potential of CBBs for both energy storage and additional functionalities, such as strain sensing enabled by conductive cement matrices. This review establishes a critical basis for future development of CBBs as multifunctional structural components in infrastructure applications.

## 1. Introduction

Modern infrastructure is increasingly expected to integrate smart functionalities alongside traditional load-bearing capabilities to support sustainability and resilience objectives [1,2,3]. Cement-based batteries (CBBs) have emerged as an innovative approach that allows concrete structures to store electrical energy and supply power to integrated electronic devices [4,5]. Modern infrastructure is increasingly expected to integrate smart functionalities alongside traditional load-bearing capabilities to support sustainability and resilience objectives [6,7,8]. This multifunctional concept aligns with the development of advanced building materials that combine structural performance with additional features such as self-sensing, thermal regulation, electromagnetic shielding, and internal energy storage [9,10,11,12]. Embedding energy storage directly within structural materials has the potential to support zero-energy or energy-positive buildings by enabling renewable energy harvesting, autonomous sensor operation, and on-site power for monitoring and maintenance systems [13,14,15,16].

**Figure 1:**
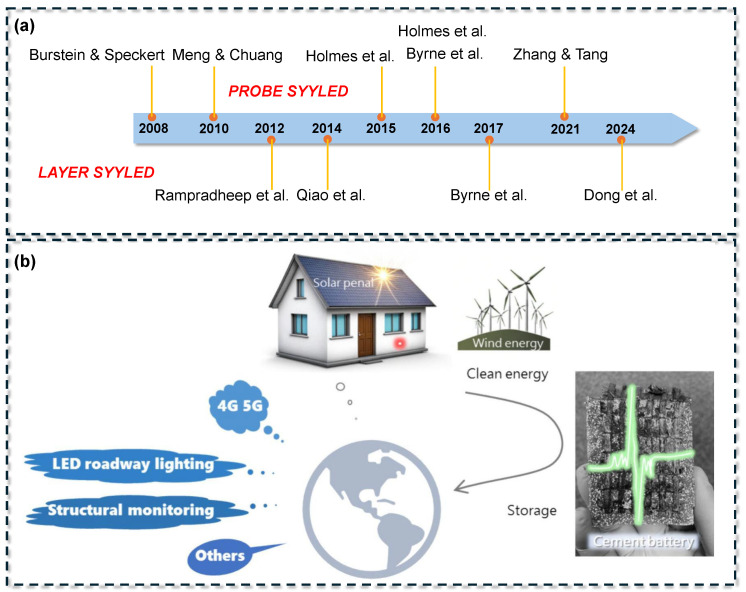
Development and conceptualization of cement-based batteries modified based on [2]: (**a**) timeline of technological milestones; (**b**) conceptual schematic illustrating CBBs for structural energy storage applications [17,18,19,20,21,22,23,24,25,26].

Early proof-of-concept studies established the fundamental feasibility of cement-based electrochemical cells, as illustrated in Figure 1. Burstein and Speckert [17] introduced a basic system comprising an aluminum anode and a steel cathode, where water reduction occurred in the cement pore solution. This configuration yielded an open-circuit voltage of approximately 0.4 V and a power density of about 10^−7^ W/cm^2^. Over the past decade, performance improvements have been achieved through advancements in material composition and cell architecture. In 2010, Meng and Chung [18] developed a layered cement-based battery incorporating dispersed zinc and manganese dioxide within the cement matrix. This design delivered an open-circuit voltage of around 0.72 V and a capacity of approximately 0.2 mAh, representing a 650% increase in current density compared to the initial prototype, although its energy density remained significantly lower than that of commercial batteries.

By 2021, Zhang and Tang [19] developed a rechargeable cement-based battery prototype with an energy density of approximately 0.8 Wh/L, equivalent to 7 Wh/m^2^ for their thin sample. This value represented a tenfold increase over earlier concrete battery designs. Although still significantly lower than the energy densities of conventional lithium-ion batteries (350–400 Wh/L) and lead–acid batteries (~10 Wh/L), the study demonstrated the feasibility of achieving rechargeability and suggested that the large volume of concrete in structural applications could compensate for the low energy density [27]. In 2024, Dong et al. [20] fabricated a cement-based triboelectric nanogenerator (TENG) using a graphene-modified cementitious composite. Their device achieved a peak power output of approximately 151.5 μW, or a power density of 95 mW/m^2^, when connected to a 100 MΩ resistor. These developments support the vision of multifunctional buildings constructed from energy-storing concrete capable of acting as large-scale battery systems. Such structures could power low-consumption devices, such as sensors, communication modules, or lighting, and provide buffering capacity for renewable energy, thereby enhancing smart grid resilience [28,29]. Additionally, these conductive cementitious materials possess intrinsic piezoresistive properties, enabling real-time structural health monitoring while simultaneously storing and supplying electrical energy [30,31].

**Figure 2:**
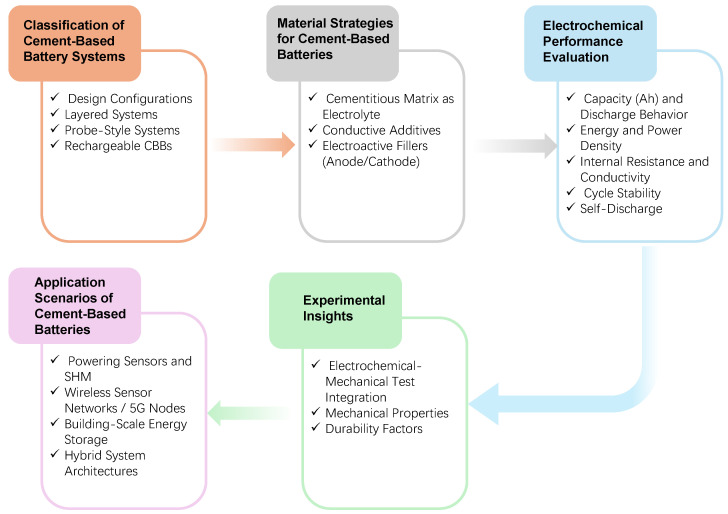
The organizational structure of this review.

Despite the rapid progress in CBB research, the literature to date lacks a dedicated, in-depth review of this topic. The present article aims to fill this gap by providing a comprehensive overview of cement-based battery systems, with particular emphasis on their dual role in structural mechanical integrity and electrochemical energy storage. In contrast to prior reviews [16,30,32] that have examined broader classes of structural energy storage materials (such as concrete supercapacitors or polymer-based structural batteries), this review is specifically on cement-based batteries and their unique challenges and opportunities. The organizational structure of the review is illustrated in Figure 2. The discussion begins with the technological background and the underlying motivation for developing CBBs. A systematic classification of design architectures is then presented, including layered monolithic systems and probe-style configurations. Material strategies aimed at improving ionic conduction, electronic transport, and redox efficiency are critically assessed. Electrochemical performance indicators, such as capacity, energy density, and cycling stability, are evaluated using data from the recent literature. Experimental approaches and major durability concerns under both environmental and mechanical influences are also reviewed. Finally, potential applications in self-powered infrastructure are discussed, and future research directions are proposed to enhance performance, scalability, and real-world applicability in civil engineering settings.

## 2. Classification of Cement-Based Battery Systems

Cement-based batteries (CBBs) demonstrate diverse structural and electrochemical configurations, reflecting the varied aims of early feasibility studies and more recent application-oriented developments. Over the past two decades, both embedded and monolithic geometries have been examined, alongside primary and rechargeable chemistries, to develop multifunctional cementitious energy systems. This section presents a systematic classification of CBBs based on design topology, electrode integration methods, and rechargeability. The progression from simple galvanic cells with dissimilar metal electrodes to layered composites incorporating engineered architectures and electrochemically active meshes indicates a marked advancement in both performance and system complexity. Table 1 outlines representative CBB systems reported in the literature, highlighting the transition from sacrificial-cell prototypes to structurally integrated, rechargeable cement-based energy storage technologies.

### 2.1. Design Configurations

**Figure 3:**
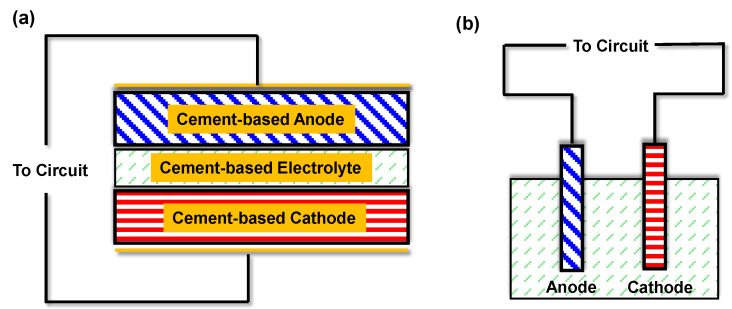
Primary system architectures developed for cement-based batteries modified based on [35] (**a**) layered (monolithic) cells and (**b**) probe-style cells.

Two primary architectural configurations have been established for cement-based batteries: layered monolithic cells and probe-style cells [35], as schematically illustrated in Figure 3. In the layered configuration, the battery consists of sequentially cast cementitious anode and cathode layers, each incorporating electroactive materials, separated by a cement-based electrolyte layer [24]. Cement serves as the continuous matrix throughout, forming an integrated composite structure after curing. The electrolyte function is fulfilled by the alkaline pore solution within the cement matrix, eliminating the need for a discrete separator as found in conventional batteries. In contrast, the probe-style configuration employs discrete metal electrodes (such as rods or plates) embedded within a bulk cementitious medium that acts as the electrolyte [36]. In this setup, the cement paste or mortar, typically contained within a structural form, mimics the behavior of an alkaline salt solution, while two dissimilar metals function as the anode and cathode [23]. The probe configuration operates as a galvanic cell embedded in concrete, whereas the layered design fully integrates the electrochemical components within the cement matrix.

In the probe-type galvanic cell (Figure 3b), the metallic anode undergoes oxidation, releasing electrons that travel through an external circuit to the cathode. Simultaneously, hydroxide ions in the cement pore solution transport ionic charge to maintain electroneutrality. This configuration operates as an embedded corrosion cell, generating current as the anode metal is gradually consumed. In contrast, the layered architecture (Figure 3a) functions as a solid-state battery embedded within the concrete. Both electrodes are integrated into the hardened matrix and facilitate complementary redox reactions, with oxidation occurring in the anode layer and reduction in the cathode layer. When connected to an external load, the layered system stores and releases charge through distributed faradaic processes, while the cement matrix serves as both an electrolyte and structural binder. The probe-type design provides single-use galvanic output determined by the metal pair’s potential difference, whereas the layered configuration allows for repeated charge–discharge cycling if reversible redox-active materials are utilized.

### 2.2. Layered Systems

The first layered CBB was developed by Meng and Chung [18], aiming to address the limitations observed in earlier probe-type prototypes. In this design, the anode, electrolyte, and cathode were all composed of cementitious materials, with ionic conduction occurring through the pore solution of the cement matrix. Zinc powder was incorporated into the cement anode layer, MnO_2_ into the cathode layer, and carbon black was added to both layers to enhance electronic conductivity. These layers, approximately 4 mm (anode), 2 mm (electrolyte), and 8 mm (cathode) in thickness, were cast sequentially into a single cement block measuring 80 × 40 × 14 mm and cured together. The system achieved an open-circuit voltage (OCV) of approximately 0.72 V and maintained a discharge of 60 µA for 3.4 h, yielding a total capacity of 0.204 mAh. Although the absolute power output was low (~1.4 µW/cm^2^), this study validated the feasibility of using cement matrices loaded with electroactive powders and conductive additives to construct functional electrochemical systems. The current density reached approximately 1.9 µA/cm^2^, around 6.5 times greater than that of the earlier Burstein and Speckert cell. This improvement was attributed to the enlarged internal interface generated by uniformly dispersing active materials within the cement matrix, which promoted ion exchange and improved utilization of electrochemical components [2,37,38].

**Figure 4:**
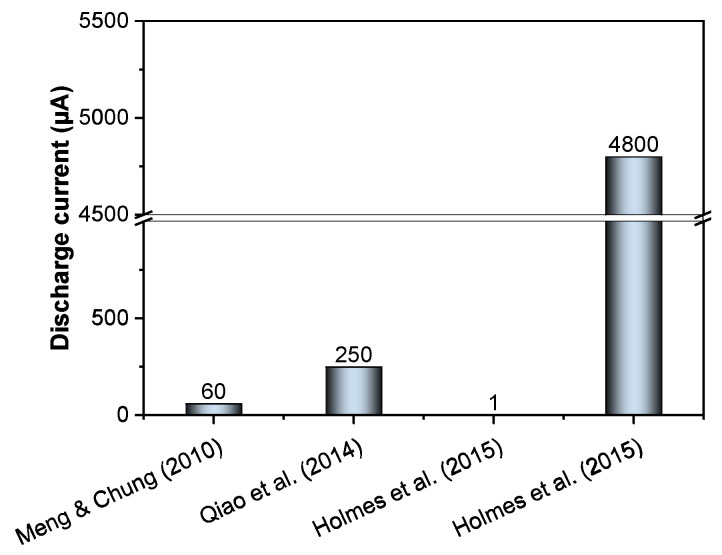
Discharge current comparison of cement-based batteries based on different refs. [18,21,22].

Subsequent research built upon this layered approach. A comparative summary of discharge performance among various CBB configurations is presented in Figure 4. Rampradheep et al. [25] fabricated a similar Zn–MnO_2_ battery (100 × 50 × 18 mm), using PEG as a curing agent to retain moisture and enhance ion transport, achieving an OCV of ~0.6. Qiao et al. [21] added carbon fibers and nanotubes to both electrodes to improve conductivity and used a cylindrical design (30 mm diameter, 70 mm height). Their cell discharged at ~250 µA for 24 h with a minimum voltage of ~0.7 V and a peak OCV of ~1.4 V. It successfully charged a supercapacitor to ~2.34 V over 32 h via a DC boost converter, showing potential for low-rate energy harvesting. Holmes et al. [22] compared layered, can-type, and probe-type designs. The layered cell showed poor performance due to internal resistance. In contrast, the can-type battery, using an aluminum container as both casing and an anode with a central copper cathode, produced an initial current of ~4.8 mA but declined quickly due to anode corrosion. This underscores a key limitation of primary CBBs: metal anodes such as Zn and Al corrode during discharge, shortening service life [39].

Beyond electrochemical performance, achieving robust interlayer bonding remains a significant challenge in layered CBBs. The successive casting of layers often results in delamination or interfacial cracking, requiring precise mix design to ensure material compatibility. Inadequate adhesion increases internal resistance and compromises durability, as electrical continuity between layers may be disrupted under mechanical or thermal stress. Therefore, the development of layered CBBs must address both electrochemical optimization and structural cohesion within the multilayer assembly.

### 2.3. Probe-Style Systems

As shown in Table 1, probe-type cement-based batteries (CBBs) consist of two metal electrodes embedded in a hardened cementitious matrix, typically a paste or mortar with high ionic conductivity. This configuration forms an alkaline electrochemical cell within the cement, analogous to a conventional galvanic cell. The open-circuit voltage (OCV) is determined by the electrochemical potential difference between the selected metals. Holmes et al. [22] examined magnesium, zinc, and aluminum as anodes paired with copper cathodes in identical cement-based electrolytes, reporting OCVs of approximately 1.34 V for Mg–Cu, 0.79 V for Zn–Cu, and 0.52 V for Al–Cu. These values correspond to the expected galvanic series in alkaline environments, with the magnesium anode producing the highest voltage, indicating its potential suitability for higher-output applications.

Byrne et al. [23,24,26] further investigated probe-type cells using aluminum or magnesium plate electrodes embedded in 90 × 90 × 40 mm mortar blocks. Measured OCVs reached approximately 1.5 V for Al–Cu and 1.6 V for Mg–Cu. Under light resistive loading, the Mg–Cu cell provided a quasi-steady current of about 0.6 mA and operated for approximately 238 h before the anode was largely depleted. These results illustrate that, although the power output of probe cells is modest, they can sustain a low-level current over extended durations, making them suitable for long-term operation in low-power applications such as microelectronic sensors or cathodic protection systems. One proposed application involves using cement-embedded galvanic cells with sacrificial magnesium anodes to deliver a corrosion prevention current while simultaneously serving as power sources [40].

Probe-type batteries are relatively simple to fabricate as they do not require distinct mix designs or the spatial layering of components [41,42]. However, they function as primary batteries; the metal anode undergoes irreversible oxidation (e.g., Zn to ZnO and Mg to Mg(OH)_2_) and cannot be readily recharged. These systems therefore act as one-time energy sources, albeit with extended lifespans under low-drain conditions. In contrast, layered CBBs offer the potential for rechargeability if appropriate reversible redox pairs are incorporated. Nevertheless, early layered designs encountered significant limitations related to low electronic conductivity and inefficient utilization of active materials, posing challenges for achieving true rechargeability [43]. In summary, probe-style CBBs offer simpler construction and higher initial output but lack rechargeability and long-term capacity. In contrast, layered CBBs trade short-term performance for rechargeability and structural integration, enabling sustained energy storage.

### 2.4. Rechargeable CBB Developments

A significant breakthrough in CBB development was reported by Zhang and Tang [33], who demonstrated a rechargeable system with improved performance. They employed a layered design incorporating several key innovations. A concentrated alkaline solution (KOH and LiOH) was added to the electrolyte layer to enhance ionic conductivity and support Ni–Fe chemistry [2,34]. Ion exchange resin particles were introduced to maintain ionic transport while inhibiting electronic conduction, functioning as a distributed separator [44,45]. Short carbon fibers replaced traditional conductive additives to form a percolating network that improved both electrical conductivity and mechanical toughness [46,47,48]. Nickel(II) hydroxide and iron powder were used as cathode and anode materials, respectively, embedded in cement layers [47]. To address electron transport limitations, electroplated Ni and Fe coatings were applied to conductive meshes, which were then cast into the cement matrix. These acted as both current collectors and electroactive material reservoirs. The resulting battery delivered over 2 mA for 22 h and a total capacity of ~88 mAh, with an average energy density of 7 Wh/m^2^ over six cycles and an OCV of ~1.8 V.

**Figure 5:**
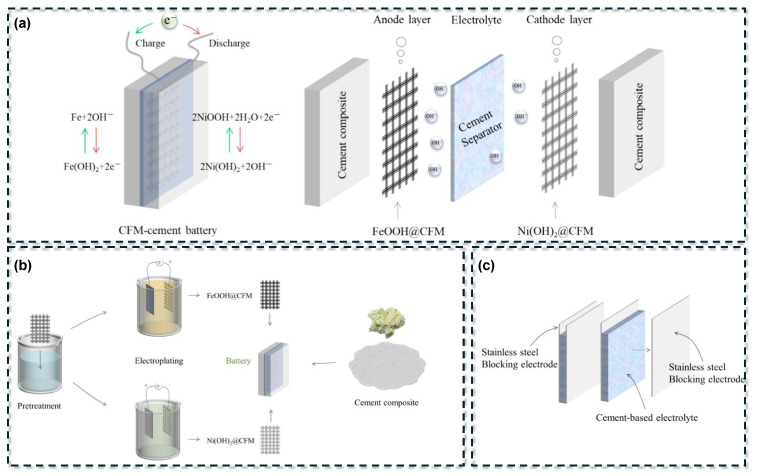
Rechargeable Ni–Fe cement-based battery configurations adapted from [34]: (**a**) cell structure with electroplated carbon fiber mesh, (**b**) fabrication process, and (**c**) ionic conductivity test setup.

Subsequent research has further improved solid-state CBB performance. Yin et al. [34] reported a similar Ni–Fe system (see Figure 5), replacing the carbon fiber mesh with a nickel foam substrate. This porous metal scaffold offers superior conductivity and a large surface area, enabling infiltration with cement slurry to form a mechanically robust and electrically efficient electrode. With Ni foam electrodes electroplated with active materials, their design achieved higher discharge capacities and energy efficiency compared to previous mesh-based systems. Their cement battery maintained stable performance over 30 cycles, achieving a peak average energy density exceeding 11 Wh/m^2^, which is the highest reported for CBBs to date. These results highlight continued progress toward closing the performance gap between CBBs and conventional rechargeable batteries.

### 2.5. Summary of Cement-Based Battery Systems

In summary, CBB systems can be categorized by structural configuration (monolithic layered versus discrete electrodes) and electrochemical type (primary versus secondary) [49]. Layered systems integrate electrochemical functionality within the concrete matrix and can be made rechargeable through appropriate material selection. In contrast, probe-type designs are simpler and suitable for single-use or maintenance-free applications such as corrosion protection, although they are not rechargeable [50,51]. Hybrid configurations have also been explored, such as those employing a cement-based electrolyte in combination with embedded reinforcing steel as one electrode and a surface layer or internal component as the counter-electrode. These approaches increasingly merge with the roles of structural components and energy systems. For example, the corrosion of steel reinforcement in saline concrete has been recognized as forming a primitive “concrete battery”, potentially capable of powering marine structural health monitoring sensors [52,53,54]. As the field progresses, the optimal design for a specific application may involve a tailored combination of structural integration, electrochemical performance, and long-term durability [55].

## 3. Material Strategies for Cement-Based Batteries

**Figure 6:**
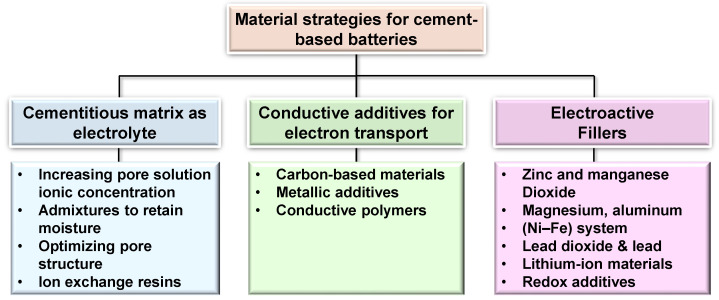
Classification of electrolytes for cement-based batteries.

The development of efficient cement-based batteries relies on the rational design of three critical material components, as illustrated in Figure 6 [56]: (1) the cementitious matrix serving as electrolyte and separator, (2) conductive additives enabling electron transport, and (3) electroactive fillers functioning as anode and cathode materials. These elements must be engineered to operate synergistically as an electrochemical system while maintaining the rheological and mechanical performance required for structural applications. This section compares material selection strategies for each component and explores methods for optimizing the overall composite system.

### 3.1. Cementitious Matrix as Electrolyte

**Figure 7:**
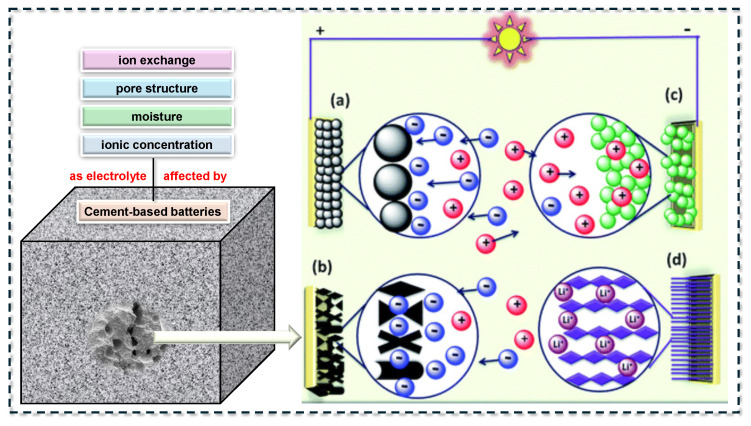
Capacitive energy storage mechanisms modified based on [59](**a**) electrical double-layer capacitance on carbon particles, (**b**) double-layer capacitance on porous carbon, (**c**) redox-based pseudocapacitance, and (**d**) intercalation-based pseudocapacitance.

Ordinary Portland cement paste or mortar is commonly used as the primary matrix in CBBs. Upon hydration, cement forms a porous solid, with its capillary pores filled by an alkaline aqueous solution (pH approximately 12–13). This pore solution contains mobile ions such as OH^−^, K^+^, Na^+^, and Ca^2+^, enabling limited ionic conduction [26,57]. Although plain hardened cement paste exhibits relatively high electrical resistivity, typically ranging from 10^5^ to 10^6^ Ω·cm, it is not a perfect insulator due to ionic transport through the connected pore network. To function effectively as a battery electrolyte, the ionic conductivity of the cement matrix must be enhanced, while its bulk electronic conductivity must remain low to prevent internal short-circuiting or self-discharge [58,59]. Several approaches to achieving this balance are discussed in the following section, and the capacitive energy storage mechanisms are also shown in Figure 7.

#### 3.1.1. Increasing Pore Solution Ionic Concentration

Ionic conductivity in cement-based systems can be significantly enhanced by incorporating salt or alkalis into the mixing water. The addition of electrolytes such as NaCl, KOH, or CaCl_2_ increases the concentration of mobile ions in the pore solution, thereby improving ionic transport [60]. Ouellette and Todd [61] reported that reinforced concrete exposed to seawater, which contains high levels of NaCl, effectively functions as an electrochemical cell through ionic conduction and corrosion processes. This phenomenon was described as a “concrete battery” capable of powering marine electronics. In CBB research, alkaline salts are commonly used to enhance electrochemical performance. For instance, Zhang and Tang [33] employed a combination of KOH and LiOH in the electrolyte layer to support Ni–Fe redox reactions, while other studies have utilized NaOH or KOH to achieve a similar effect. However, excessive salt content can interfere with cement hydration and adversely affect early-age strength development [62].

#### 3.1.2. Admixtures to Retain Moisture

The effectiveness of the pore solution depends on the retention of sufficient liquid water within the hardened cement matrix [63,64]. Drying significantly reduces ionic mobility, thereby impairing conductivity. To mitigate this, self-curing agents or hydrophilic polymers such as polyethylene glycol (PEG) and polyvinyl alcohol (PVA) are often incorporated to enhance moisture retention. In one study, PEG was added to maintain long-term electrolyte hydration, thereby improving the stability of electrochemical output [23]. Polyethylene oxide (PEO), a component of solid polymer electrolytes, has also been investigated for its ability to increase ionic conductivity while reducing water loss, effectively forming a polymer–cement composite electrolyte [65,66]. Recent research demonstrated that incorporating PEO along with lithium and potassium salts into the cement matrix substantially enhanced discharge capacity, likely by preserving continuous ionic pathways during hydration. The gel-forming behavior of PEO in the pore solution may support sustained ion transport as the matrix matures [67,68]. Recent research demonstrated that incorporating PEO along with lithium and potassium salts into the cement matrix substantially enhanced discharge capacity, likely by preserving continuous ionic pathways during hydration. The gel-forming behavior of PEO in the pore solution may support sustained ion transport as the matrix matures.

#### 3.1.3. Optimizing Pore Structure

A higher water-to-binder (W/B) ratio generally increases the porosity and connectivity of pores, enhancing ionic conductivity at the expense of mechanical strength [69,70,71]. This creates a trade-off: adequate porosity is essential for ion transport, but excessive porosity can compromise structural integrity. For CBB electrolytes, moderate W/B ratios (typically between 0.4 and 0.5) are commonly adopted to ensure a continuous ionic pathway while maintaining sufficient strength. The incorporation of fine supplementary cementitious materials (SCMs), such as fly ash, silica fume, or slag, can further refine the pore structure and improve moisture retention. For example, silica fume has been reported to enhance pore uniformity and has been combined with carbon black to balance electrical conductivity and mechanical performance. In addition to OPC-based systems, alkali-activated binders such as geopolymers have been investigated for use as CBB electrolytes [72,73,74,75]. These materials inherently possess high concentrations of alkali ions, contributing to improved ionic conductivity. Although still relatively underexplored, geopolymer-based batteries present a promising alternative due to their high ion mobility and lower environmental impact compared to the conventional Portland cement system [76,77,78].

#### 3.1.4. Ion Exchange Resins

As demonstrated by Zhang and Tang [33], particulate ion exchange resins incorporated into cement can function as nanoscale separators. These resins permit ionic transport while inhibiting electron flow, thereby increasing internal resistance and reducing electronic leakage currents [79]. Typically composed of sulfonated polymers, the resins swell in the presence of electrolytes and create rapid ion transport pathways. When uniformly dispersed within the cement matrix, they divide the pore structure into micro-domains separated by electronically insulating regions, which helps suppress self-discharge [80]. This strategy was effective in maintaining a stable open-circuit voltage in the Ni–Fe system, even under high KOH concentrations, without causing internal short-circuiting between electrodes [81].

### 3.2. Conductive Additives for Electron Transport

Unlike metals or graphitic electrodes in conventional batteries, plain hydrated cement is a poor electronic conductor (its matrix is essentially an insulator with only the pore fluid conducting ions) [17]. To facilitate electron transport within cement-based electrodes, conductive admixtures must be incorporated. These additives establish a percolating network of conductive pathways within the hardened composite.

#### 3.2.1. Carbon-Based Materials

**Figure 8:**
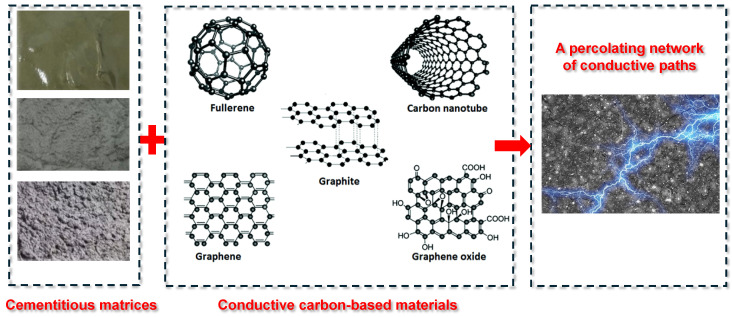
Schematic illustration of conductive network formation in cement-based composites using carbon-based materials modified based on [85].

Carbon-based additives are widely used in CBBs due to their stability and electrical conductivity [82,83,84,85], as illustrated in Figure 8. Carbon black (CB), in particular, has been extensively applied to reduce the resistivity of cementitious matrices [86]. Adding a small amount of fine CB (particle size ~50 nm) can lower resistivity by several orders of magnitude once the percolation threshold is reached. Meng and Chung [18] used 0.5% CB by weight of cement, while later studies increased this to 1–2% to ensure network formation. Carbon fibers (CFs), including microfibers and nanofibers, are also effective due to their high aspect ratio, enabling connectivity at lower volume fractions [87,88]. Dosages of 0.5–1% CF can significantly enhance conductivity while improving matrix toughness. Zhang and Tang [33] identified 0.5% CF as optimal for balancing conductivity and workability.

Other conductive additives such as carbon nanotubes (CNTs), graphene nanoplatelets, and graphene oxide have shown promise for bridging microcracks and contributing pseudocapacitance [82]. However, their dispersion requires surfactants or ultrasonication to avoid agglomeration. Hybrid strategies combining CB with carbon nanofibers have been proposed to leverage multi-scale conduction pathways [89]. Coarser fillers like graphite powder and coke breeze have also been explored but require higher loading [90]. The goal remains to form a continuous carbon network within the electrode layer. Excessive carbon, however, can dilute the cement and active material content, compromising both mechanical integrity and energy storage capacity [2,6]. Therefore, optimizing the type, size, and dosage of carbon additives is essential to achieve effective performance with minimal trade-offs.

#### 3.2.2. Metallic Additives

**Figure 9:**
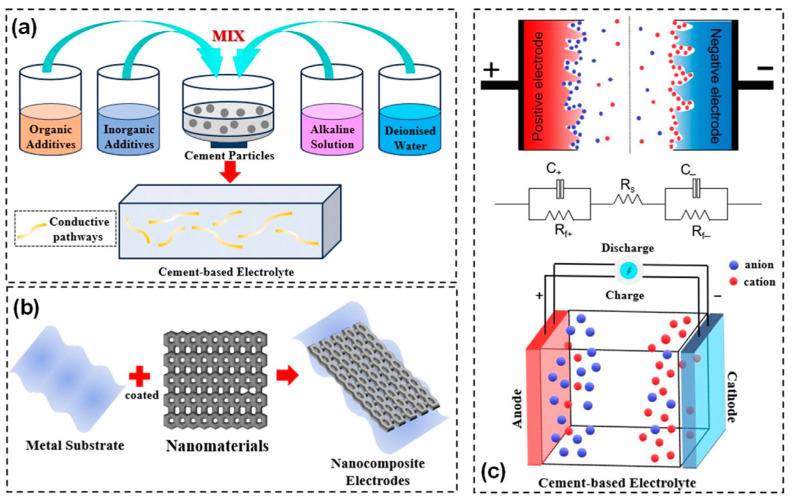
Cement-based supercapacitor configurations adapted from [94]: (**a**) electrolyte preparation, (**b**) electrode fabrication, and (**c**) energy storage mechanism.

Metallic particles or fibers such as steel, iron, nickel, and copper have also been employed to enhance electrical conductivity in cement-based systems [91,92,93,94], as shown in Figure 9. Steel fibers, for example, have been used to simultaneously provide structural reinforcement and electrical conduction in smart concretes [95]. However, in battery applications, the selection of metal additives requires caution to prevent undesired electrochemical reactions. Metals with electrochemical potentials differing from the intended electrodes may undergo corrosion or electroplating during charge–discharge cycles [96,97]. To address this, inert conductive metals such as nickel and stainless steel powders have been explored. In some studies, iron and nickel powders were incorporated not only as conductive agents but also as active materials [98]. Metals typically offer higher electrical conductivity per unit volume than carbon-based additives. However, their greater density and the potential for galvanic corrosion present challenges if not properly managed [99]. Moreover, surface passivation, such as the formation of oxide layers on iron, can diminish conductivity over time, which is an issue generally avoided with carbon-based materials.

#### 3.2.3. Conductive Polymers

**Figure 10:**
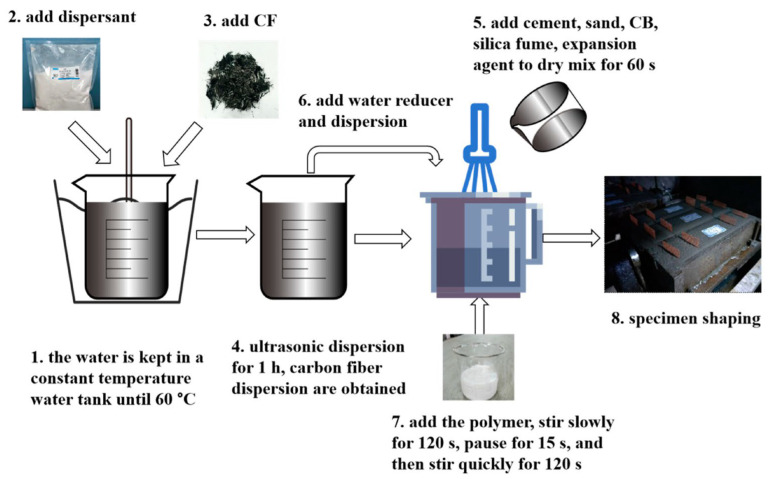
Mixing and casting process of polymer-modified, carbon-fiber-reinforced cementitious composites for electrical applications [101].

Intrinsically conducting polymers such as polypyrrole, polyaniline, and PEDOT:PSS have the potential to improve the electrical conductivity of cementitious materials [100,101,102], an application of which is shown in Figure 10. In cement-based batteries, such polymers may not only facilitate electron transport but also contribute to charge storage through pseudocapacitive behavior [103]. However, concerns remain regarding their long-term stability in the highly alkaline cement pore environment, and their application in CBB electrodes remains limited.

Balancing electrical conductivity, active material content, and mechanical strength is critical for performance optimization [104]. A continuous conductive network is necessary to ensure electron transport to all electroactive regions, while excessive carbon content may increase porosity or disrupt hydration due to particle agglomeration. To address this, many studies optimize mix proportions by measuring composite resistivity across different filler dosages to identify the percolation threshold. A slightly higher dosage than the threshold is typically selected to achieve conductivity without compromising workability or structural integrity [105]. For example, Zhang and Tang [33] evaluated various carbon fiber contents and dispersion methods, including the use of surfactants, to determine a formulation with low resistivity and good workability. The incorporation of silica fumes has also been found to counteract the reduction in strength caused by high carbon content by improving the interfacial bond between carbon and the cement matrix. Previous studies have shown that mixtures containing both silica fume and carbon black exhibit better mechanical properties than those using carbon black alone [106,107].

### 3.3. Electroactive Fillers (Anode/Cathode Materials)

**Figure 11:**
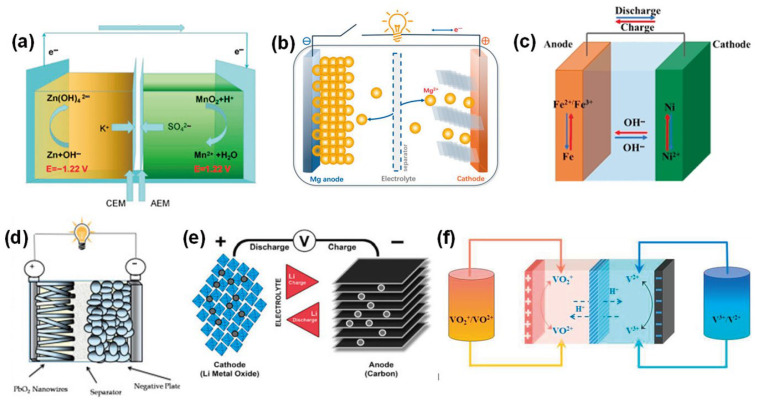
Electroactive material configurations for cement-based batteries: (**a**) alkaline Zn/MnO_2_ [108], (**b**) Mg-based high-voltage system [109], (**c**) Mg-based high-voltage system [110], (**d**) PbO_2_/Pb analog [111], (**e**) Li-ion intercalation framework [112], and (**f**) Li-ion intercalation framework [32].

Electroactive materials, which undergo redox reactions to store and release energy, serve as the core components of battery functionality [32,108,109,110,111,112]. In cement-based batteries, electrodes can be categorized as either composite electrodes, where active particles are dispersed within the cement matrix, or bulk electrodes, consisting of solid metal elements such as steel rebars [113]. Most layered cement-based batteries employ composite electrodes incorporating powdered active materials uniformly distributed in the cement paste. Figure 11 summarizes the application of various electroactive fillers in such systems.

#### 3.3.1. Zinc (Anode) and Manganese Dioxide (Cathode)

The Zn/MnO_2_ pair was widely employed in early cement-based battery studies, replicating the chemistry of primary alkaline batteries [108]. Zinc metal powder functions as the anode, undergoing oxidation to ZnO in the alkaline pore solution of cement, while MnO_2_ in the cathode layer is reduced from Mn(IV) to Mn(III) when accepting electrons [114]. The redox reactions in the cement matrix are analogous to those in conventional alkaline batteries:

Anode:     Zn + 2 OH^−^ → ZnO + H_2_O + 2 e^−^ (1)

Cathode:     2 MnO_2_ + H_2_O + 2 e^−^ → Mn_2_O_3_ + 2 OH^−^ (2)

These batteries benefit from the low cost and wide availability of Zn and MnO_2_, both of which exhibit stability in alkaline environments. Zinc tends to form a passivating ZnO layer that remains within the cement matrix [113]. The overall capacity is constrained by the quantity of active materials incorporated into the system. Meng and Chung [18] adopted an anode-to-cathode mass ratio of 0.5, accounting for the differing consumption rates of Zn and MnO_2_. Qiao et al. [21] introduced a high concentration of these powders, particularly in a 50 mm thick cathode layer enriched with MnO_2_. However, certain limitations persist. Zinc corrosion can generate hydrogen gas, as described by the reaction Zn + 2H_2_O → Zn(OH)_2_ + H_2_, potentially leading to internal voids or pressure buildup. Additionally, MnO_2_ is inherently insulating and requires the formation of an effective conductive network to be electrochemically active. The incorporation of carbon materials into the mix helps address this challenge by establishing conductive pathways, thus forming Zn/carbon and MnO_2_/carbon composite electrodes.

#### 3.3.2. Carbon-Based Materials Magnesium and Aluminum (Anodes)

These metals have been utilized in probe-type cells for high-voltage primary batteries [109]. Magnesium, in particular, offers a high driving voltage (approximately 1.3 to 1.6 V versus copper) and a high theoretical capacity [115]. However, both magnesium and aluminum anodes are susceptible to continuous corrosion in the alkaline environment of cement, producing oxides or hydroxides. This leads to self-discharge over time, even in the absence of an external electrical load. The resulting corrosion products, such as Mg(OH)_2_ or Al(OH)_3_, may provide some benefit by filling pores within the cement matrix, as they exhibit cementitious properties. Nevertheless, the accumulation of these products eventually passivates the electrode surface, thereby inhibiting further electrochemical reactions [116]. Magnesium and aluminum anodes are therefore more suitable for short-term applications requiring higher voltage over limited durations, such as in emergency sensing systems. These materials are not rechargeable in cement-based systems, as reversing the hydroxide formation to redeposit metallic Mg or Al would demand impractically high charging voltages. In addition, aluminum readily forms complex aluminate species in highly alkaline environments, further limiting reversibility [117]. Despite these limitations, sacrificial Mg or Al electrodes remain attractive for low-power, self-sustained sensing applications due to their simplicity and high initial energy output.

#### 3.3.3. Nickel–Iron (Ni–Fe) System

The combination of nickel hydroxide (Ni(OH)_2_) and iron powder, first applied in rechargeable CBBs by Zhang and Tang [33], forms a stable and efficient redox pair for alkaline systems. The primary reactions are as follows: Fe + 2 OH^−^ ⇌ Fe(OH)_2_ + 2 e^−^ at the anode, and Ni(OH)_2_ + OH^−^ ⇌ NiO(OH) + H_2_O + e^−^ at the cathode, both proceeding forward during discharge. This electrochemical system operates at a nominal voltage of approximately 1.2 to 1.4 V. Known for its long cycle life and overcharge tolerance, the Ni–Fe couple is particularly appealing for use in cement-based energy storage [110].

In the cement matrix, both iron and nickel hydroxide require effective conductive networks to ensure full utilization. Iron oxidation produces Fe(OH)_2_ or FeO, which may not consistently maintain electrical contact with the conductive pathways. To address this, Zhang and Tang [33] adopted an electroplating strategy in which Ni(OH)_2_ and Fe were deposited onto separate conductive meshes. This approach facilitated strong electronic connectivity between the active materials and the current collectors. The success of this system demonstrates that rechargeable chemistries can be effectively implemented in cement-based batteries, provided the electroactive components are properly integrated [118]. Other alkaline rechargeable systems, such as Ni–Zn or Ag–Zn, may also be considered, although zinc presents challenges due to its high solubility in alkaline environments [119]. The choice of Ni–Fe was likely influenced by the fact that both discharge products, Fe(OH)_2_ and NiOOH/Ni(OH)_2_, remain solid and relatively immobile within the cement matrix.

#### 3.3.4. Lead Dioxide and Lead (PbO_2_/Pb)

A lead–acid-type system can be conceptually adapted for cement-based applications by using PbO_2_ as the cathode and Pb or powdered Pb as the anode [111,120]. In a highly alkaline environment, lead would be converted to plumbate species, while PbO_2_ would remain relatively stable. Although this configuration has not been extensively studied, primarily due to the toxicity of lead, its implementation is theoretically feasible. Nevertheless, embedding a miniature lead–acid system within concrete raises significant environmental concerns, particularly in scenarios involving structural damage or demolition.

#### 3.3.5. Lithium-Ion Materials

An intriguing concept involves integrating high-energy lithium-ion battery materials into a structural cementitious matrix [112]. For instance, cathode materials such as LiFePO_4_ or LiMn_2_O_4_, paired with compatible anodes like graphite or LiTiO_2_, could theoretically be embedded within cement or alkali-activated systems [121,122]. However, lithium-ion batteries typically require non-aqueous or solid electrolytes, as the presence of water in conventional cement matrices leads to rapid degradation of lithium-containing materials. Lithium metal or lithiated carbon will react with water to form LiOH, hydrogen gas, and other by-products, rendering them incompatible with hydrated cement environments [123,124]. While a dry cement composite incorporating solid polymer or ceramic-based lithium electrolytes may offer a potential solution, such designs fall outside the scope of conventional concrete technology and remain largely experimental [125,126]. To date, no successful demonstration of a lithium-based cement battery has been reported, and the approach remains impractical with currently available materials. Consequently, research has predominantly focused on aqueous-based chemistries that are more compatible with cementitious environments, including alkaline systems and analogues such as Zn–air or Ni–Cd.

#### 3.3.6. Redox Additives

In addition to conventional electrode fillers, several studies have introduced redox-active additives into cement matrices to enhance charge storage capacity [32,127]. Conductive metal oxides such as RuO_2_ and MnO_2_ have been investigated for their pseudocapacitive properties and considered for use in cement-based supercapacitors [128]. Organic redox compounds, including ferrocene and quinone derivatives, may also contribute to faradaic capacitance if properly encapsulated to prevent leaching. A recent approach employed nano-sized Fe_2_O_3_ and NiCo_2_Sₓ coatings on nickel foam embedded within cement, effectively forming integrated faradaic electrodes within the composite structure [128]. These advanced materials offer a hybrid energy storage mechanism that bridges the functions of batteries and capacitors, delivering higher power output while introducing greater material and processing complexity.

### 3.4. Summary of Material Strategies

Optimizing the electrode composition in CBBs requires achieving continuous ionic and electronic pathways. The uniform dispersion of all components is essential, as the agglomeration of conductive additives or active particles can result in electrically isolated regions and reduced efficiency [82]. Techniques such as ultrasonication or the use of dispersants (e.g., surfactants for carbon and citric acid for metal hydroxides) are commonly employed. The ratio between active materials and conductive fillers must also be balanced. Insufficient conductive additives hinder charge transport, while excessive content dilutes the active phase and may compromise mechanical properties [46]. Percolation thresholds are typically determined by monitoring bulk resistivity. In parallel, chemical compatibility with cement hydration must be considered [129,130]. Percolation thresholds are typically determined by monitoring bulk resistivity. In parallel, chemical compatibility with cement hydration must be considered [131], and alkaline additives like KOH or LiOH may shift hydration behavior, producing by-products such as K_2_SO_4_ or Li_2_CO_3_ [132,133]. These effects can be mitigated by incorporating supplementary cementitious materials (e.g., slag or fly ash) to improve chemical stability and mechanical performance. The use of fine SCMs like silica fume also helps offset strength loss caused by non-cementitious fillers such as MnO_2_ [134,135].

The ideal CBB formulation combines a cement matrix that provides mechanical support and ionic conductivity, a well-distributed conductive network for electron transport, and electroactive materials capable of stable, reversible redox reactions [2,136]. Achieving this integration requires balancing conductivity, reactivity, and structural integrity. Experimental studies have shown that tailored composites, such as systems incorporating cement, carbon fibers, KOH electrolytes, and ionomer resin, along with Ni(OH)_2_ or Fe and embedded conductive meshes, can simultaneously store energy and bear mechanical load [2,33]. Although most current demonstrations rely on simple Portland cement formulations with limited additives, future advancements are expected to include nano-engineered materials, modified active surfaces, and precision processing techniques such as 3D printing. These interdisciplinary approaches will be essential to realizing high-performance, multifunctional cement-based energy storage systems.

## 4. Electrochemical Performance Evaluation

The performance of cement-based batteries is evaluated through a series of electrochemical parameters, similar to those used for conventional batteries but adapted to the characteristics of cementitious systems. Key metrics include capacity, energy density, power density, internal resistance (or conductivity), cyclic stability, and self-discharge rate [137]. This section defines these metrics within the context of cement-based batteries, summarizes representative performance values, and outlines the measurement methodologies commonly employed.

### 4.1. Capacity (Ah) and Discharge Behavior

Battery capacity refers to the total charge delivered from full charge to cutoff voltage, typically expressed in ampere-hours (Ah) or milliampere-hours (mAh) [138], as shown in Equation (3):

C = I × t(3)

where III is the discharge current (A) and t is the discharge time (h). In CBBs, capacity is commonly measured by galvanostatic discharge, using low currents (microampere to milliampere range) due to limited power output. For example, a cell developed by Chung delivered 60 µA for 3.4 h (0.204 mAh), while Zhang and Tang’s Ni–Fe system achieved >2 mA for ~22 h (88 mAh total) [33]. Typical capacities range from 5 to 50 mAh for a 100 × 100 mm cell. Although results are often reported per cell, comparisons across systems may use capacity density (e.g., mAh/kg), though standardization remains limited due to diverse cell architectures.

Unlike conventional batteries, CBBs often exhibit a continuous voltage decline during discharge rather than a stable plateau. This behavior results from polarization effects due to internal resistance and the nonuniform distribution of electroactive sites within the cement matrix. For instance, Qiao et al. [21] observed an initial open-circuit voltage of approximately 1.4 V that dropped rapidly to 0.7 V under load and continued to decrease gradually over 24 h. Similarly, Holmes et al. [22] reported current spikes in probe cells followed by rapid decay, indicating high internal resistance and the fast depletion of accessible charge. The low utilization of active materials is a persistent limitation in CBBs, as a significant portion of embedded electroactive particles may remain electrically isolated or become passivated. Enhancing material utilization remains a key research objective, with strategies focusing on conductive scaffolds, improved particle dispersion, and optimized electrode architecture.

### 4.2. Energy Density

Energy density refers to the amount of energy stored per unit mass or volume and is a key metric for comparing performance with conventional batteries [139]. It is calculated as Equation (4):

(4) $$E=\frac{C\times V_{ave}}{m}\;\mathrm{or}\;E=\frac{C\times V_{ave}}{V}$$

where E is energy density (Wh/kg or Wh/m^3^), C is capacity (Ah), V_ave_ is average discharge voltage, and m or V represents the battery’s mass or volume. In commercial systems, energy densities range from 60 to 100 Wh/L for lead–acid batteries to 250 to 700 Wh/L for lithium-ion technologies. In contrast, cement-based batteries exhibit significantly lower values. For example, the 2021 Chalmers system achieved ~0.8 Wh/L, while Dong et al. [140] reported similar results even with ionic additives and optimized electrodes. Yin et al. [2] recently demonstrated a Ni–Fe cement-based battery reaching approximately 1.0–1.5 Wh/L, based on an estimated 11 Wh/m^2^ for a 1 cm thick cell.

Although these values are orders of magnitude below those of lithium-ion cells, the large volume of concrete in infrastructure offers compensatory storage potential. A structure containing 100 m^3^ of active concrete material at 1 Wh/L could theoretically store 100 kWh, sufficient to power lighting or sensor networks. This “scale compensates density” approach is often cited to justify the concept. Nonetheless, improving intrinsic energy density remains essential. Strategies include increasing active material content, adopting higher-voltage chemistries, and improving the utilization of theoretical capacity. Incorporating pseudocapacitive elements such as NiOOH or conductive polymers may further enhance energy storage by enabling electrostatic charge accumulation within the cement matrix.

### 4.3. Power Density and Internal Resistance

Power density (W/kg or W/L) indicates the rate at which a battery can deliver energy [4,141,142]. It is calculated as Equation (5):

(5) $$P_{ed}=\frac{P_{max}}{m}$$

where P_ed_ is the power density, P_max_ is the maximum power output, and m is the battery mass. CBBs typically exhibit a low power density due to high internal resistance. This resistance stems from multiple sources: ionic conduction limitations in the pore solution, electronic resistance within the composite (especially below the percolation threshold), interfacial charge transfer resistance, and diffusion constraints in the pore structure.

Electrochemical impedance spectroscopy (EIS) is commonly employed to assess these resistive components. For instance, Yin et al. [34] reported significantly reduced impedance in systems using nickel foam electrodes compared to carbon mesh, resulting in higher achievable power. Nevertheless, most CBBs can only sustain discharge currents in the microampere to milliampere range, corresponding to output powers in the microwatt to milliwatt range. This is insufficient for high-drain applications. Zhang and Tang’s [33] Ni–Fe cell, for example, reached an estimated peak power of approximately 2.4 mW (2 mA × 1.2 V) over an 8 cm^2^ cell area (~0.3 mW/cm^2^), far below the tens of mW/cm^2^ delivered by standard lithium-ion coin cells. One of the highest observed outputs was reported by Holmes et al. [22], where an Al–Cu cell briefly produced 19 mA at ~0.7 V (~13 mW), although the effect was short-lived.

The intrinsic electrical conductivity of cement-based electrodes typically ranges from 10^−3^ to 10^0^ S/m, significantly lower than that of aqueous electrolytes (~10^2^ S/m) or metals (>10^4^ S/m), suggesting ample opportunity to reduce internal resistance. The incorporation of nickel foam substantially improves electronic pathways and provides a robust current collector, enabling higher current output with reduced polarization [143]. Future evaluations of power performance should involve discharge profiling across multiple current levels and characterization via EIS to identify limiting mechanisms. Enhancing power density is essential for enabling short-duration, high-power functions such as operating sensors or wireless transmitters. At present, however, CBBs remain more suitable for low-power, continuous operation scenarios.

### 4.4. Cycle Life and Stability

Rechargeable CBBs remain at an early stage, with limited data on cycle life. Zhang and Tang [33] reported six stable cycles, likely constrained by test duration rather than degradation. Yin et al. [2] achieved 30 cycles with minimal capacity loss. These results are promising but remain well below commercial standards. Degradation in CBBs may result from several factors [1,5,15]. First, active material may lose contact with the conductive network due to volume changes or phase transitions, such as between Ni(OH)_2_ and NiOOH. The cement matrix offers some mechanical restraint, but repeated cycling can cause microcracking or disrupt percolation, especially if corrosion-induced expansion occurs. Second, the dissolution of active ions like Zn^2+^ or Ni^2+^ may lead to permanent loss. Third, local pH shifts or the formation of soluble species such as Fe(OH)_4_^2−^ can affect the cement chemistry over time. Fourth, while carbon additives are generally stable, high anodic potentials may trigger degradation through oxygen evolution, though most CBBs operate below this threshold.

Cycle testing must accommodate slow charge kinetics and high internal resistance, often requiring long charging at low currents [1,5,15]. Techniques such as cyclic voltammetry and potentiostatic holds are used to identify redox behavior and control charging. Coulombic efficiency remains a key metric. In aqueous systems, Ni–Fe typically shows moderate efficiency (50–70%), which may be further reduced in cement due to side reactions. Although specific values are rarely reported, efficiency can be inferred by comparing charge and discharge capacities. Strategies such as sealing and catalyst integration may help mitigate gas evolution and improve stability.

### 4.5. Self-Discharge

Self-discharge refers to the spontaneous loss of charge in a battery over time without an external load [144]. In CBBs, this phenomenon may result from internal short circuits, such as electronic leakage through unintended conductive pathways between electrodes, or from parasitic reactions like anode corrosion induced by residual oxygen in the pores. The use of ion-exchange resins and insulating admixtures aims to limit electronic conduction and reduce such leakage. Properly constructed layered CBBs, with physically separated electrodes, typically exhibit high internal resistance to direct electron flow, thereby minimizing self-discharge.

However, probe-type cells containing continuous electrolytes are more susceptible to galvanic self-discharge, particularly if external moisture or embedded conductive components, such as interconnected steel rebars, complete the circuit. Few studies report long-term open-circuit voltage (OCV) retention, but some have observed gradual OCV decay over several days, likely due to anode oxidation by residual oxidants or leakage currents through the moist cement matrix [145,146]. The accurate quantification of self-discharge requires extended OCV monitoring. Potential mitigation strategies include partial drying of the cell to limit ionic mobility or the introduction of physical separators, such as polymer films, to better isolate electrode layers.

### 4.6. Summary of Testing and Characterization Methods

**Figure 12:**
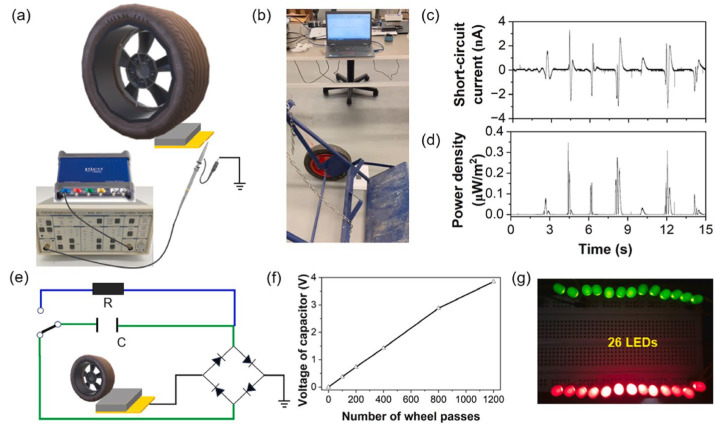
Experimental demonstration of a cement-based triboelectric nanogenerator (TENG) under wheel loading, adapted from Dong et al. [147]: (**a**) schematic and (**b**) photograph of the test setup; (**c**) short-circuit current and (**d**) power density output; (**e**) circuit design for capacitor charging; (**f**) voltage evolution of a 10 μF capacitor under repeated wheel passes; (**g**) illumination of 26 LEDs powered by the charged capacitor.

Standard battery characterization methods are commonly adapted for CBBs, often using embedded wire leads or surface-mounted current collectors, as illustrated in Figure 12 [147]. Current CBBs exhibit a moderate performance, typically delivering capacities in the tens of milliampere-hours, energy densities below 2 Wh/L, and low power output. Despite these limitations, such a performance is notable given the structural and functional constraints. It is already adequate for low-duty-cycle sensors or emergency lighting. Ongoing improvements in materials and design continue to enhance output. To ensure meaningful comparisons, standardized performance reporting, such as normalization per unit volume or cement mass, is needed. In parallel, systematic studies on how mechanical factors like cracking or stress affect electrochemical behavior will be critical for advancing integration with structural sensing applications.

## 5. Experimental Insights on Electrochemical–Mechanical Testing and Durability

Cement-based batteries straddle the domains of electrochemistry and structural engineering [148,149]. Therefore, experimental evaluations must address both electrochemical performance and mechanical integrity, often simultaneously. In this section, we discuss the key experimental methods used to characterize CBBs on both fronts, insights gained from these tests, and the durability considerations unique to this multifunctional technology.

### 5.1. Electrochemical Testing in Cementitious Specimens

Measuring the electrochemical behavior of batteries embedded in concrete presents greater challenges than conventional cells, primarily due to difficulties in electrolyte containment, maintaining stable electrode contact, and the extended timescales involved [150]. Various experimental setups have been adapted to address these issues.

#### 5.1.1. Embedded Electrodes and Instrumentation

**Figure 13:**
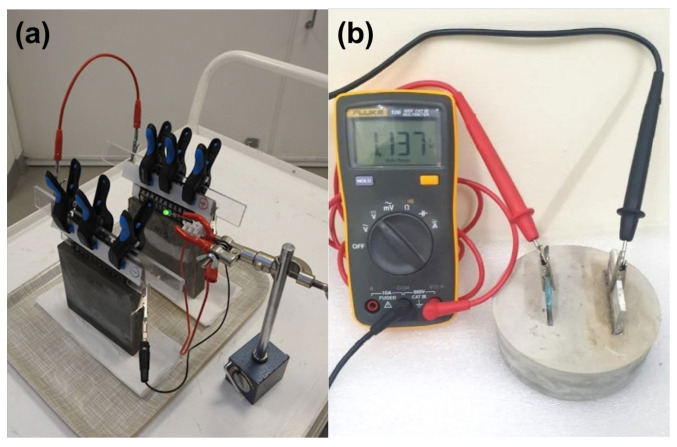
Cement-based battery experimental setup modified based on [46]: (**a**) prototype configuration of the cement-embedded battery; (**b**) open-circuit voltage measurement.

In layered CBB samples, metal current collectors such as wire mesh or foil are typically embedded within the electrode layers to serve as terminals for charge and discharge. Reliable electrical contact is essential; in most cases, the mesh is connected to an external copper wire that is insulated from the cement matrix except at the designated connection point [5]. In probe-style configurations, leads are attached directly to metal probes embedded in the concrete, as illustrated in Figure 13 [46]. To prevent unintended galvanic interactions, insulating coatings or sealants are applied to exposed lead wires, limiting contact with the cement electrolyte to the intended electrode interfaces.

#### 5.1.2. Containment

Some experiments cast cement batteries in molds that also function as containers (for instance, using PVC tubes for cylindrical cells, as shown in Figure 13 b) to prevent moisture loss and mimic a sealed cell environment [151]. This also allows filling any void space above the cement with an electrolyte solution if needed (though most prefer fully solid systems).

#### 5.1.3. Multifunctional Loading Frames

**Figure 14:**
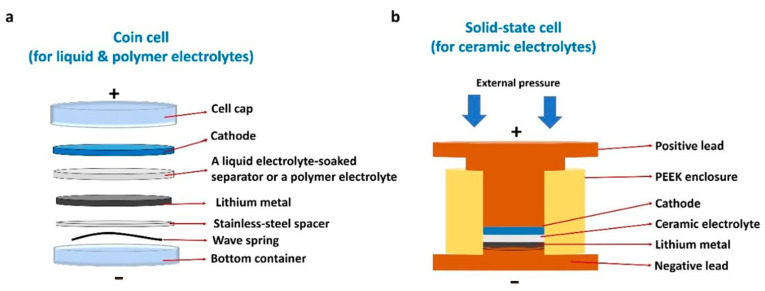
Custom experimental setups for studying mechanical–electrochemical coupling in cement-based batteries, adapted from [152]: (**a**) coin-cell type system for electrolyte screening; (**b**) solid-state configuration used to evaluate electrode performance under stress.

To investigate the coupling between mechanical loading and electrochemical behavior, customized experimental setups have been employed [87,88]. A representative configuration is shown in Figure 14 [152]. For instance, a conductive cement prism, serving as a battery electrode, can be positioned within a uniaxial loading frame equipped with electrical contacts to monitor resistance or galvanic current under applied compressive or tensile stress. Similar configurations are widely used in smart concrete studies to assess resistivity changes under mechanical load. In the context of cement-based batteries, discharge tests can be conducted concurrently with mechanical loading to evaluate how stress influences the discharge profile. Although limited studies have explored this coupling, it is increasingly recognized as essential for validating structural health monitoring performance under realistic service conditions, such as discharging a cement battery embedded in a flexural beam subjected to cyclic loading.

#### 5.1.4. Environmental Control

Cement-based battery samples are highly sensitive to moisture and temperature. Electrochemical testing is often conducted shortly after curing while pore moisture remains present, as performance typically declines upon drying [83]. To preserve moisture stability, some studies enclose specimens in plastic film or maintain them in humid environments during testing. Temperature influences both ionic conductivity and reaction kinetics. Most tests are performed at ambient conditions (approximately 20–25 °C), although selected experiments have explored performance under lower (e.g., 0 °C) or elevated temperatures (e.g., 40 °C) to assess potential degradation or increased self-discharge. While limited data are available on the temperature dependence of cement-based batteries, their behavior is anticipated to resemble that of aqueous battery systems, exhibiting reduced kinetics at low temperatures and accelerated degradation at higher temperatures.

### 5.2. Mechanical Property Testing

Because cement-based batteries are designed to serve structural functions, their mechanical properties, including compressive strength, tensile or flexural strength, and elastic modulus, must be evaluated to ensure compliance with structural requirements. The addition of conductive or electroactive fillers may influence these properties and should be carefully assessed.

#### 5.2.1. Compressive Strength

**Figure 15:**
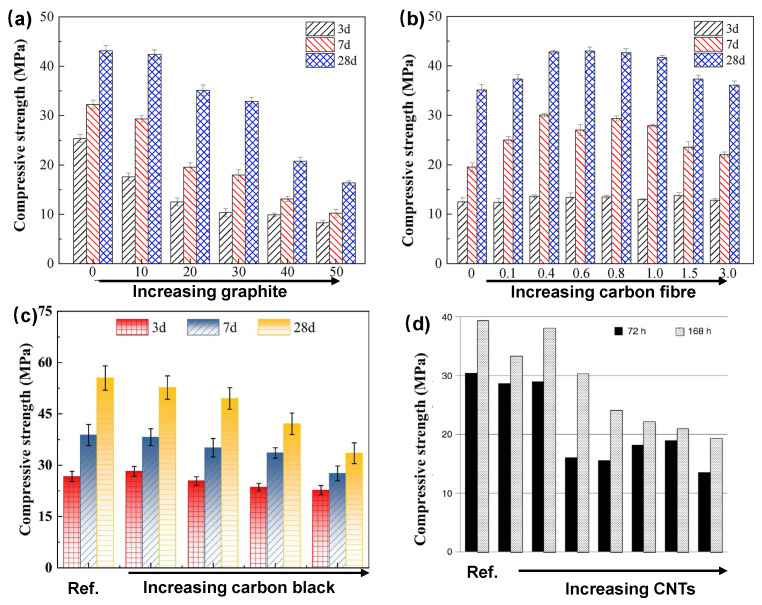
Influence of carbon-based additives on the compressive strength of cement composites: (**a**) graphite [48], (**b**) carbon fiber [48], (**c**) carbon black [153], and (**d**) carbon nanotubes [23].

In general, the incorporation of non-structural fillers such as carbon or metal powders tends to slightly reduce compressive strength. This reduction is primarily attributed to the dilution of the cementitious matrix and the possible formation of voids or weak interfaces [23,48,153], as illustrated in Figure 15. For instance, carbon black possesses a high surface area and may interfere with cement hydration if not adequately dispersed, potentially leading to strength loss. However, when added in moderate amounts (typically less than 1% by weight), its impact is often minimal, with several studies reporting only a minor decrease in compressive strength in well-formulated carbon-filled systems. In some cases, carbon fibers have been found to enhance flexural or tensile strength by bridging microcracks, although their influence on compressive strength is generally neutral or slightly adverse unless incorporated at high volumes. In the study by Zhang and Tang [33], the use of short carbon fibers at 0.5% volume did not significantly affect compressive strength. Instead, the fibers contributed to improved toughness and a reduction in shrinkage-induced cracking.

Conversely, incorporating large amounts of metal powders, such as 10% zinc, or polymeric materials can significantly reduce mechanical strength. Byrne et al. [23], while optimizing cement-based batteries for cathodic protection, identified a formulation that balanced electrical conductivity with sufficient compressive strength, suggesting that empirical adjustment was necessary to maintain structural performance above 30 MPa. In applications involving non-load-bearing components, such as facade panels, higher additive content may be acceptable despite a reduction in strength. However, for load-bearing elements, such as structural columns, the proportion of additives must be limited to preserve the required strength classification.

#### 5.2.2. Flexural and Tensile Behavior

Conductive fibers such as carbon or steel can enhance post-cracking performance by promoting more ductile failure, which is advantageous for structural applications [47,48,150]. Their piezoresistive properties also enable the early detection of microcrack formation before visible damage occurs, making them suitable for structural health monitoring. Standard mechanical tests, including three-point bending and split-cylinder tensile tests, have been applied to sensing concretes to correlate mechanical loading with an electrical response. A notable increase in electrical resistance typically coincides with initial cracking, serving as an early warning of structural distress. In cement-based batteries embedded within structural elements, such resistance changes can be monitored as indicators of damage initiation. Despite the presence of conductive additives, the primary load-bearing capacity remains dependent on the cement matrix and the incorporated fiber reinforcement [150]. To meet structural performance requirements, sufficient fiber content should be incorporated, whether through traditional steel reinforcement, carbon-based conductive fillers, or a combination of both.

#### 5.2.3. Bond and Interface

When embedded electrodes such as mesh or metal foam are used, the interfacial bond between the electrode and the surrounding cement matrix is critical for both mechanical integrity and electrical performance. Pullout tests are often conducted to evaluate the anchorage strength of the mesh. Zhang and Tang [33] reported that the nickel-coated carbon mesh was fully embedded in the cement and functioned similarly to reinforcement. However, if the cement matrix lacks sufficient conductivity, current is primarily carried through the mesh itself. Mechanically, slippage or interfacial corrosion should be avoided. Surface treatments, such as roughening or applying a conductive polymer coating, have been shown to enhance bonding.

#### 5.2.4. Carbon-Based Materials Shrinkage and Creep

The incorporation of polymers or large volumes of metal can influence drying shrinkage and creep behavior in cement-based materials. Carbon fibers have been shown to mitigate shrinkage by restraining the matrix, which is particularly beneficial for thin battery layers, as shrinkage-induced cracking may compromise structural integrity and disrupt electrical conductivity [154]. In this context, fibers function as both reinforcement and conductive pathways. While long-term creep under sustained loading has not been extensively examined for cement-based batteries, increased porosity or the presence of inclusions is likely to elevate creep due to matrix softening [155]. This factor should be accounted for when CBB elements are intended for prolonged load-bearing applications.

### 5.3. Durability Testing

Durability refers to the long-term performance of CBBs when subjected to environmental stressors and mechanical fatigue throughout their service life [156]. Key considerations include the effects of moisture, temperature fluctuations, chemical exposure, and cyclic loading.

#### 5.3.1. Cycle Stability (Mechanical)

Structures are subjected to numerous load cycles, such as traffic-induced stress on bridges or wind-induced movement in buildings [82,157]. CBBs intended for such environments must retain both electrical and mechanical functionality under repeated loading. Piezoresistive concretes have demonstrated a reasonable fatigue performance, with only minor signal degradation after extensive cycling, typically caused by microcrack development or fiber damage. These materials generally withstand hundreds of thousands of cycles within the elastic range [87]. When combined with electrochemical cycling, a coupled electro-mechanical fatigue scenario arises. To date, no studies have reported the long-term testing of CBBs under simultaneous mechanical loading and charge/discharge cycling. Repeated electrode expansion and contraction during metal plating and stripping, when coupled with mechanical stress, may accelerate degradation. Mitigation strategies could include designing flexible interfaces or incorporating strain-tolerant fiber electrodes.

#### 5.3.2. Environmental Exposure

Cementitious materials are subject to environmental stressors such as temperature fluctuations, moisture cycling, freeze–thaw conditions, and chemical exposure (e.g., de-icing salts or carbonation) [156]. In CBBs, freeze–thaw cycles may induce internal cracking that disrupts the conductive network. Battery function may also be impaired by moisture redistribution, as frozen pore solutions lose ionic conductivity, rendering the battery inactive at subzero temperatures until thawing occurs [158]. The inclusion of antifreeze admixtures, such as salts already present in many CBBs, can help depress the freezing point. High humidity or immersion may enhance ionic conductivity but also increase the risk of electrode corrosion in the presence of oxygen. Long-term carbonation from CO_2_ exposure gradually reduces pH, which can alter the electrochemical stability of active materials. For example, reduced alkalinity may affect zinc dissolution or compromise the Ni–Fe redox couple, which operates optimally in strongly alkaline environments. Maintaining alkaline conditions in the inner layers may require protective outer coatings or sufficient concrete cover to mitigate surface carbonation [33,113].

#### 5.3.3. Chemical Stability

The constituent materials in a CBB must exhibit long-term chemical stability and must not degrade the surrounding concrete matrix [159,160]. For instance, the introduction of KOH can trigger an alkali–silica reaction (ASR) when reactive aggregates are present, potentially causing expansion and cracking over time. To prevent this, inert aggregates should be selected, or supplementary cementitious materials such as fly ash or metakaolin may be incorporated to bind excess alkalis and mitigate ASR risk [161,162,163]. Additionally, embedded steel components, such as reinforcement bars or steel fibers, must be protected from corrosion. Although conventional concrete environments maintain steel passivation through high alkalinity, steel incorporated into the electrochemical circuit may undergo anodic polarization, resulting in intentional corrosion, as observed in sacrificial anodes [164]. While acceptable for designated sacrificial elements, this is unsuitable for primary reinforcement. Therefore, design approaches may involve isolating structural steel from battery circuits, using corrosion-resistant alloys or applying protective coatings. Alternatively, if reinforcement is intentionally integrated into the battery function, it must be ensured that the corrosion rate remains low enough to preserve structural integrity throughout the service life.

### 5.4. Summary of Landscape for CBBs

In essence, the experimental framework for CBBs combines electrochemical testing with structural performance evaluation. Current evidence highlights the necessity of optimizing both electrical and mechanical properties at the same time. Although long-term durability under service conditions is still being explored, initial results are promising. With appropriate material choices such as stable nickel and iron chemistries, inert carbon-based conductive networks, and protective admixtures, CBBs can operate reliably over multiple charge–discharge cycles. They are also capable of supporting structural loads while exposed to environmental conditions [1,140]. Future research is expected to include long-term field monitoring, accelerated aging tests through temperature and moisture cycling, and combined mechanical and electrochemical experiments to assess full lifecycle performance within structural applications.

## 6. Applications in Self-Powered Infrastructure

**Figure 16:**
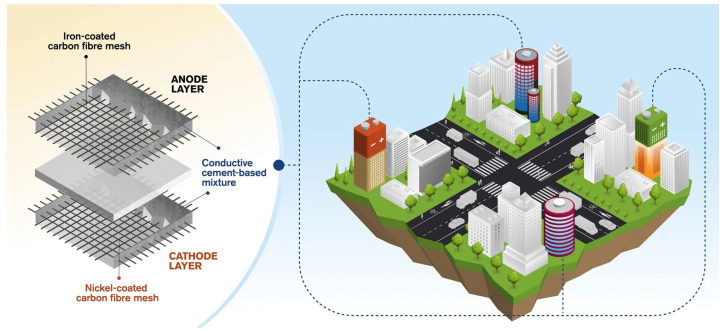
Schematic of a cement-based battery cell incorporating metal-coated carbon fiber mesh electrodes and its envisioned application in energy-storing buildings, modified based on [169].

The integration of energy storage and self-sensing capabilities in CBBs enables a wide range of innovative applications in civil infrastructure [157,165,166,167]. These multifunctional systems have the potential to convert traditionally passive structural components into active elements that can supply power, monitor structural health, and interface with broader energy networks [10,168]. A conceptual illustration of this approach is provided in Figure 16, which depicts a cement-based battery incorporating metal-coated carbon fiber mesh electrodes and its prospective deployment in energy-storing buildings. This section outlines both current developments and future prospects for CBB applications in intelligent, self-sustaining infrastructure systems, including bridges, buildings, and roadways.

### 6.1. Self-Powered Structural Health Monitoring

In addition to serving as power sources for sensors, CBBs can also operate as sensors themselves, as discussed in the previous section. This dual functionality enables self-monitoring concrete components. For example, a self-sensing pavement could detect vehicular loads and structural damage while simultaneously harvesting energy from mechanical vibrations or solar input [140,147]. One notable development is the use of cement-based triboelectric nanogenerators (TENGs) to convert traffic-induced vibrations into electrical energy. Peng et al. [170] demonstrated that a 40 mm × 40 mm cement-based TENG was capable of producing an open-circuit voltage of approximately 100 V, sufficient to charge a capacitor and power a sensor. When deployed at scale, a roadway embedded with multiple CBB or TENG units could support functions such as weigh-in-motion systems or crack detection by monitoring changes in electrical resistance. In military or security contexts, an instrumented concrete surface could detect impacts or foot traffic without relying on external power sources, using energy from the detected event to generate a signal. Although still in early stages, these technologies demonstrate significant potential for autonomous structural monitoring.

### 6.2. Wireless Sensor Networks and 5G Infrastructure

The development of 5G networks and smart city infrastructure is expected to result in the widespread deployment of small cells and distributed sensors [87,171]. Many of these devices may be mounted on or integrated into concrete structures to enhance durability. CBBs offer a potential solution by providing embedded power sources within the structural material itself. Researchers at Chalmers University have proposed using concrete-based batteries to support 4G connectivity in remote areas. This concept could be extended to applications such as self-powered concrete fence posts functioning as communication repeaters. Similarly, edge computing devices for monitoring environmental parameters or traffic conditions could be embedded in concrete poles and operated using structural energy storage systems.

### 6.3. Large-Scale Energy Storage (Vision for the Future)

A long-term objective in CBB research is the utilization of entire buildings or structural foundations as grid-connected energy storage systems [172]. If the energy density of CBBs can be moderately improved, the large volume of concrete used in infrastructure could be harnessed for distributed storage. For instance, the concrete foundations of wind turbines could store excess wind-generated energy, or building envelopes across a district could collectively serve as a decentralized battery network to stabilize renewable power supply fluctuations [94]. This concept, sometimes referred to in the media as “power concrete,” envisions mass energy storage integrated directly into structural materials. While its feasibility depends on addressing technical challenges and ensuring economic competitiveness with conventional battery systems, the approach is appealing because it embeds energy storage within existing infrastructure, avoiding the need for additional space or materials.

**Figure 17:**
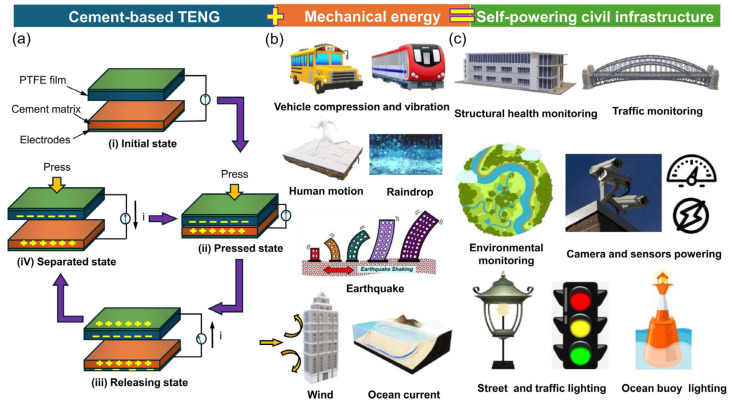
Cement-based TENG for infrastructure applications adapted from [20]: (**a**) triboelectric mechanism, (**b**) sources of mechanical energy, and (**c**) example applications in smart infrastructure.

Dong et al. [20] illustrated several potential applications (shown in Figure 17) in a smart city setting. Their proposed configurations include concrete batteries powering electric vehicle (EV) charging stations, streetlights, and residential loads. For example, sidewalks or parking areas constructed from battery-integrated concrete panels could wirelessly charge EVs parked or moving overhead. Such applications would require rapid power delivery, potentially achieved through a hybrid system combining supercapacitive discharge for fast charging with gradual recharging from the grid during idle periods. Similarly, solar-powered streetlights with battery-embedded concrete bases could store energy during the day and operate autonomously at night. While these concepts remain visionary, they offer a valuable direction for ongoing research and development.

### 6.4. Structural Considerations in Applications

When integrating CBBs into structural elements, it is essential to evaluate the interaction between electrochemical functionality and mechanical performance [173]. One strategy involves designating specific layers or zones within a component to serve as the active battery region, while the remaining sections are optimized for load bearing. A practical configuration is the sandwich panel, consisting of outer layers composed of high-performance structural concrete and an inner core containing the battery material. This layered approach allows for the partial decoupling of mechanical and electrochemical functions: the external layers resist bending stresses, while the inner core facilitates energy storage and sensing. Such composite panels may be applied in facades or flooring systems, contributing to a building’s distributed energy network. Additionally, retrofitting existing infrastructure is feasible through the application of thin cement-based battery coatings, which function as overlays to endow legacy structures such as bridges or buildings with self-sensing and energy storage capabilities.

## 7. Conclusions and Future Perspectives

Cement-based battery technology remains at an early developmental stage, and its successful implementation in real-world infrastructure requires addressing several limitations and advancing interdisciplinary knowledge. This section outlines the primary challenges currently limiting CBB application and identifies key research directions needed for future development. Emphasis is also placed on the integration of CBBs with emerging technologies such as nanomaterials, artificial intelligence, and renewable energy systems to facilitate their transition from experimental prototypes to functional components within smart infrastructure.

### 7.1. Conclusions

Cement-based batteries present a promising pathway for integrating energy storage into structural materials, thereby enabling multifunctional and self-powered infrastructure. Despite growing academic attention and a body of experimental investigations, CBB technology remains in an early developmental phase. Based on a critical review of materials, mechanisms, and applications, the following conclusions are drawn:

(1) CBBs currently demonstrate relatively low energy and power densities compared to conventional electrochemical systems. This limitation is primarily attributed to sluggish electrode kinetics and the intrinsically low ionic conductivity of cementitious electrolytes.
(2) The majority of studies rely on Zn–Mn or Zn–Fe redox systems, which exhibit issues such as electrode passivation, low utilization efficiency, and rapid capacity degradation. A broader exploration of high-performance and corrosion-resistant electrode chemistries is urgently required.
(3) Although aqueous electrolytes such as NaOH and KOH improve initial conductivity, they are susceptible to leaching, carbonation, and pore blockage, which collectively undermine long-term durability.
(4) The electrochemical interfaces between electrodes and the cement matrix are typically nonuniform and characterized by high internal resistance. Research into tailored surface treatments and engineered interfacial layers remains limited but holds significant potential.
(5) The multifunctional integration with structural health monitoring is conceptually promising but experimentally underdeveloped. Although piezoresistive behavior and electrical responses under mechanical stress have been observed, the concurrent optimization of electrochemical and mechanical sensing performance remains insufficient.
(6) A lack of standardized performance metrics, including open-circuit voltage, impedance, and cycle life, limits meaningful cross-comparison and benchmarking across studies.
(7) Most experimental investigations focus on small-scale laboratory cells with limited structural relevance. Large-scale demonstrators embedded in realistic infrastructure settings are notably absent.
(8) Critical parameters for real-world applications, such as energy delivery capacity and service life under operational loads, are often overlooked, highlighting a disconnect between academic research and engineering practice.
(9) While CBBs utilize relatively low-cost materials, trade-offs involving performance limitations, fabrication complexity, and uncertain long-term durability pose challenges for large-scale deployment.
(10) The integration of advanced tools such as artificial intelligence for material design, machine learning for sensing interpretation, and hybridization with triboelectric or solar technologies remains largely untapped, constraining system-level innovation.

Realizing the full potential of CBBs requires a comprehensive and interdisciplinary research strategy encompassing electrochemistry, materials engineering, structural mechanics, and data-driven optimization. Scaling up from proof-of-concept to infrastructure-integrated systems will necessitate overcoming material performance bottlenecks, improving interface stability, and developing scalable manufacturing techniques. The incorporation of nanomaterials, wireless monitoring capabilities, and self-healing functionalities may ultimately transform CBBs into viable components of next-generation intelligent infrastructure.

### 7.2. Future Perspectives

The advancement of CBBs hinges on several critical breakthroughs that could accelerate their practical deployment:

(1) The development of high-performance electrode materials capable of significantly enhancing energy density without compromising the integrity of the cement matrix. This may involve novel composites or nano-engineered particles.
(2) The establishment of mechanisms to preserve ionic conductivity under dry conditions, potentially through self-humidifying electrolytes or polymer-integrated systems.
(3) The demonstration of reliable rechargeability and long-term cycling stability, ideally maintaining electrochemical performance over hundreds of cycles to validate multi-year operational lifespans in structural applications.
(4) The real-world integration of sensing and energy storage capabilities in a unified system, demonstrating that both functionalities can operate concurrently without mutual interference.
(5) Scalable production methods for conductive concrete that ensure consistency (perhaps an AI-controlled batching system or a factory precast approach where conditions are tightly controlled).

In summary, while considerable technical challenges remain, the development trajectory of CBBs points toward increased multifunctionality and broader integration into infrastructure systems. This emerging technology brings together electrochemistry, materials science, and structural engineering to realize the vision of intelligent, energy-active civil structures [174].

Future progress will require a sustained interdisciplinary effort, informed by both experimental evidence and advanced simulation tools. Artificial intelligence is expected to play a growing role in material design, process optimization, and data interpretation. The next decade will likely witness the first full-scale demonstration projects incorporating CBB components, offering critical insights into long-term performance and feasibility.

The success of CBBs will ultimately depend on their ability to deliver tangible benefits such as enhanced energy resilience, reduced maintenance costs, and added functionality, without introducing prohibitive complexity or expense. If these objectives are met, the impact may be transformative: infrastructure elements may evolve from passive supports into active, responsive systems that store energy and monitor structural health. This paradigm shift would redefine the role of construction materials in future smart cities, supporting sustainability, efficiency, and resilience in the built environment. The foundational research being conducted today is laying the essential groundwork for this transformation.

## Figures and Tables

**Table 1:** Representative CBB systems showing structural types, electrode compositions, and performance evolution.

Type	Electrodes and Key Additives	Output and Performance	Ref.
Probe (Al–steel)	Al anode; steel cathode; cement electrolyte	~0.4 V; ~100 nW/cm^2^ power	[17]
Layered	Zn powder anode; MnO_2_ cathode; C black	0.72 V OCV; 0.2 mAh capacity; 1.4 µW/cm^2^	[19]
Layered (cyl)	Zn/MnO_2_ + C fiber/CNT; PEG in electrolyte	~0.7–1.4 V; ~35 µA/cm^2^; charged supercapacitor	[21]
Probe/Can	Mg or Zn or Al anodes; Cu cathode	Mg–Cu: 1.3 V OCV (0.1 mA load); Can: ~5 mA initial	[22]
Probe (Mg–Cu)	Mg anode plate; Cu plate	1.6 V OCV; ~0.6 mA for 238 h (primary cell)	[23]
Layered (Ni–Fe)	Ni(OH)_2_ cathode; Fe anode; CF mesh + KOH/resin	1.8 V OCV; >2 mA for 22 h; 7 Wh/m^2^; rechargeable	[33]
Layered (Ni–Fe)	Ni foam + rGO/NiCo_2_Sₓ cathode; Ni foam + Fe_2_O_3_ anode; resin	~1.5 V(est.); >11 Wh/m^2^ over 30 cycles; improved kinetics	[34]

## Data Availability

No new data were created or analyzed in this study.

## Abbreviations

The following abbreviations are used in this manuscript:

CBB	Cement-Based Battery
SHM	Structural Health Monitoring
TENG	Triboelectric Nanogenerator
OCV	Open-Circuit Voltage
CNT	Carbon Nanotube
CF	Carbon Fiber
CNF	Carbon Nanofiber
CB	Carbon Black
SEM	Scanning Electron Microscopy
EDS	Energy-Dispersive X-ray Spectroscopy
EIS	Electrochemical Impedance Spectroscopy
OPC	Ordinary Portland Cement
SCM	Supplementary Cementitious Material
ASR	Alkali-Silica Reaction
AI	Artificial Intelligence
PEG	Polyethylene Glycol
PVA	Polyvinyl Alcohol
KOH	Potassium Hydroxide
NaOH	Sodium Hydroxide
LiOH	Lithium Hydroxide
MnO_2_	Manganese Dioxide
Mn_2_O_3_	Manganese(III) Oxide
Ni(OH)_2_	Nickel(II) Hydroxide
NiOOH	Nickel Oxyhydroxide
Fe(OH)_2_	Iron(II) Hydroxide
LiFePO_4_	Lithium Iron Phosphate
LiMn_2_O_4_	Lithium Manganese Oxide
LiTiO_2_	Lithium Titanate
PbO_2_	Lead Dioxide
CV	Cyclic Voltammetry
EV	Electric Vehicle
